# The African swine fever control zone in South Africa and its current relevance

**DOI:** 10.4102/ojvr.v83i1.1034

**Published:** 2016-05-23

**Authors:** Noluvuyo R. Magadla, Wilna Vosloo, Livio Heath, Bruce Gummow

**Affiliations:** 1 Department of Agriculture and Rural Development, Johannesburg, South Africa; 2 Department of Production Animal Studies, University of Pretoria, South Africa; 3 CSIRO-Australian Animal Health Laboratory, Geelong, Australia; 4 Agricultural Research Council, Onderstepoort Veterinary Institute, South Africa; 5 Discipline of Veterinary Science, James Cook University, Australia

## Abstract

African swine fever (ASF) has been reported in South Africa since the early 20th century. The disease has been controlled and confined to northern South Africa over the past 80 years by means of a well-defined boundary line, with strict control measures and movement restrictions north of this line. In 2012, the first outbreak of ASF outside the ASF control zone since 1996 occurred. The objective of this study was to evaluate the current relevance of the ASF control line as a demarcation line between endemic ASF (north) areas and ASF-free (south) area and to determine whether there was a need to realign its trajectory, given the recent outbreaks of ASF, global climate changes and urban development since the line’s inception. A study of ASF determinants was conducted in an area 20 km north and 20 km south of the ASF control line, in Limpopo, Mpumalanga, North West and Gauteng provinces between May 2008 and September 2012. The study confirmed that warthogs, warthog burrows and the soft tick reservoir, *Ornithodoros moubata*, are present south of the ASF control line, but no virus or viral DNA was detected in these ticks. There appears to be an increasing trend in the diurnal maximum temperature and a decrease in humidity along the line, but the impact of these changes is uncertain. No discernible changes in minimum temperatures and average rainfall along the disease control line were observed between 1992 and 2014. Even though the reservoirs were found south of the ASF boundary line, the study concluded that there was no need to realign the trajectory of the ASF disease control line, with the exception of Limpopo Province. However, the provincial surveillance programmes for the reservoir, vector and ASF virus south of this line needs to be maintained and intensified as changing farming practices may favour the spread of ASF virus beyond the control line.

## Introduction

African swine fever virus (ASFV) is a highly contagious DNA *arbovirus* belonging to the genus *Asfivirus* of the family *Asfarviridae* (Fauquet *et al.* 2005) affecting domestic pigs. The virus replicates in both the mammalian host and *Ornithodoros moubata* complex ticks (also called tampans), the arthropod host (Dixon *et al.* 2004). The infection is characterised by high morbidity and mortalities of up to 100% in domestic pigs, but the presence of the disease can remain unnoticed in wild pigs, with neonatal common warthogs (*Phacochoerus africanus*) developing viraemia high enough to infect *Ornithodoros* ticks that feed on them (Bastos *et al.* 2009; Thomson 1985). The virus can also cause mortalities in ticks (Kleiboeker & Scoles 2001).

The virus is maintained and transmitted through different cycles including the following: (1) the typical sylvatic cycle, where the ASFV is maintained between warthogs and tampans with occasional spill-over to domestic pigs, (2) the endemic cycle (tampan or domestic pig cycle) which has been reported in East Africa and (3) the domestic cycle, involving the domestic pig population where ASFV can be transmitted by direct contact between infected and susceptible domestic pigs. The common warthog is the preferred vertebrate host for *Ornithodoros* ticks that inhabit preexcavated burrows used for farrowing and shelter (Arnot, Du Toit & Bastos 2009). Together the warthogs and ticks are the determinants of the sylvatic cycle for maintenance and transmission of African swine fever (ASF) in the South African context (Magadla 2015).

**FIGURE 1:**
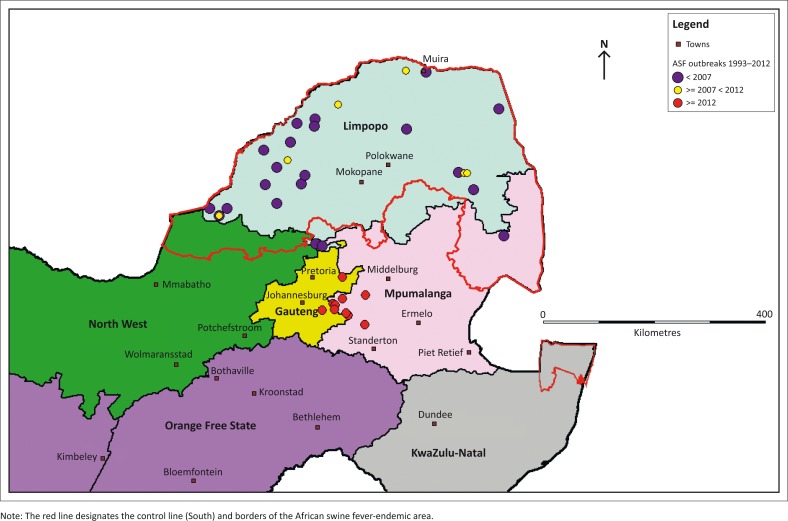
Spatial distribution of African swine fever outbreaks in South Africa between 1993 and 2012.

In South Africa, reports of ASF date back to as early as 1926 when it was first recorded in the northern parts of the country, formerly known as Transvaal (Boshoff *et al.* 2007). In 1935, South Africa instituted and gazetted a designated ASF control area that mainly encompasses the Limpopo Province, the northern parts of North West and KwaZulu-Natal provinces and the north-eastern parts of Mpumalanga Province (Figure 1). The designation of the area was based on the presence of epidemiologically significant factors (i.e. Host, Environmental and Agent factors) and the presence of outbreaks (Penrith, Thomson & Bastos 2004). The last reported outbreak in Mpumalanga occurred in 1951. In 1996, an outbreak was reported just outside the control area in Bela-Bela, Limpopo Province (Penrith *et al.* 2004). During this period, a number of cases or outbreaks occurred within the control zone in the Limpopo Province and were reported to the Department of Agriculture, Forestry and Fisheries (DAFF), South Africa, and subsequently to the World Organisation for Animal Health (OIE).

In January 2012, Gauteng Veterinary Services reported to DAFF a suspected case of ASF in a group of pigs that demonstrated clinical signs at a Gauteng abattoir. This was the first outbreak of ASF outside the control zone since 1996 (Gauteng Veterinary Services 2012). Within a period of 2 months from the confirmation of the index case, South Africa reported an additional 16 outbreaks of ASF to the OIE, all diagnosed and confirmed outside the ASF-controlled area in Gauteng and Mpumalanga provinces (OIE 2014). The spatial distribution of the ASF outbreaks that occurred in South Africa between 1993 and 2012 is shown in Figure 1.

The article describes the first study to investigate the relevance of the ASF control line as a demarcation between endemic ASF (north) areas and ASF-free (south) areas, since its institution. It also examines available climatic data along the control line to assess if there have been changes in climatic factors between 1993 and 2012 that could influence warthog or tampan distribution.

## Materials and methods

### Study area

**FIGURE 2:**
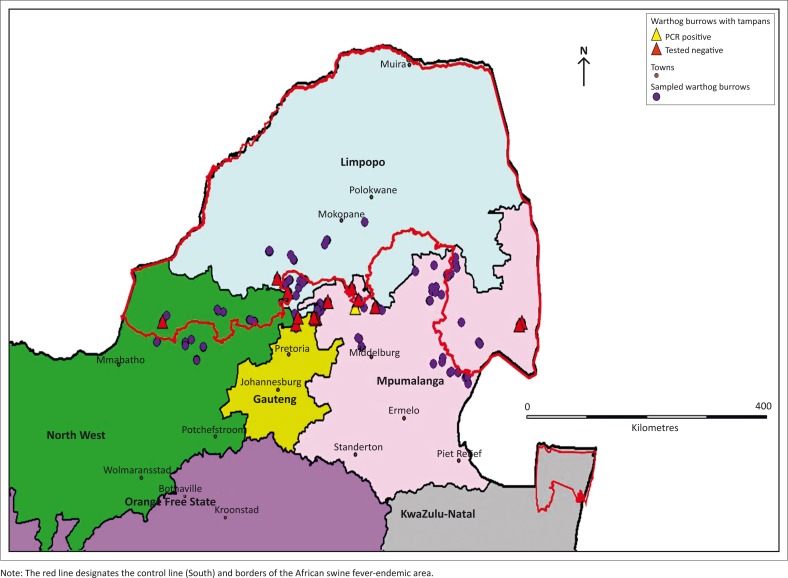
Sampling area (between yellow lines) and spatial distribution of warthog burrows sampled for presence of tampans.

The ASF control line, as determined by the *Animal Diseases Act* *of South Africa* (Act 35 of 1984), was used as a reference for the study area. Using the ASF control line as a basis, a 20-km virtual boundary was built both north and south of the control line to traverse Limpopo, Mpumalanga, North West and Gauteng provinces (Figure 2). The ASF control area in KwaZulu-Natal Province was not included because of distance and limited resources. A sampling frame of farms in the study area was compiled using area maps obtained from the Department of Water Affairs, Pretoria, South Africa, and the list of farms obtained from Limpopo and Gauteng Provincial offices of DAFF. The total number of farms in the sampling frame was 1575. All samples were collected between May 2008 and September 2012. The aim of the survey was to establish the ASFV distribution pattern along the control line. This would give an indication on whether the line served as a boundary for the disease in historic and recent outbreaks.

### Survey design

The survey was designed to sample 61 warthog burrows (i.e. the sampling unit was burrows and not farms). This number was based on the assumption that 20% of warthog burrows had infected tampans (Pretorius *et al*. 2004) and the sample size was calculated using the formula:

$$n=1.96^{2}\frac{P(1-P)}{d^{2}}$$ [Eqn 1]

where *n* is the sample size, *P* is prevalence of warthog burrows with infected tampans and *d* = 10% is the margin of error at a 95% confidence interval (Thrusfield 2005).

The study was based on the assumption that 20 in every 100 farms had warthog burrows (Pretorius *et al*. 2004) and using the formula *N* = *n* + Negative Binomial (*n* + 1, *p*), where *n* is the number of farms needed to be found and *p* is the proportion with warthog burrows with infected tampans (Vose 2001). To be 95% confident of finding 61 burrows, 304 farms needed to be sampled. Proportional weighting, based on the total number of farms in each province, was used to determine the number of farms to be sampled in each province. The selection of the 304 farms from the sample frame was carried out using Survey toolbox, Random Village sampling (Cameron 1999). Warthog burrows were purposefully selected on each farm according to whether they were recently used by warthogs.

### Warthog burrow sampling

Farms were visited between May and November during 2008–2012, and with the aid of farm staff warthog burrows were identified on each of the farms surveyed. Each burrow was scraped 10 times using a spade specially modified for this purpose, spending a minimum of 30 min and a maximum of 45 min per burrow. Scraping followed a set pattern of two scrapings each in the proximal (entrance) area, the deep areas, each of the sides and the bottom. A black plastic sheet was spread next to the burrow. The collected soil scrapings were spread on the black sheet under direct sunlight to facilitate detection of the tampans. All collected tampans were submitted to a central submission point, the Onderstepoort Veterinary Institute, Onderstepoort, Gauteng, South Africa, as part of their Transboundary Animal Diseases Programme.

### Detection of African swine fever virus DNA in tick samples

At the Onderstepoort Veterinary Institute, total DNA was extracted from a pool of tampans crushed in a 1.5 mL Eppendorf tube containing 1 mL of phosphate-buffered saline, supplemented with 1% foetal calf serum and 1% of a combination of antibiotics and an antimycotic. Each pool of tampans was composed of tampans from the same warthog burrow. Homogenates were centrifuged at 10 000 x g for 1 min and the supernatant frozen at -70 °C. DNA was extracted from 200 µL of each tick homogenate and recovered in a final volume of 50 µL DNA solution using the Qiamp kit (Qiagen GmbH, Hilden), according to the manufacturer’s instructions. A nested polymerase chain reaction (PCR) that targets the C-terminal end of the *p72* gene was used to screen soft tick samples for the presence of ASFV DNA (Basto *et al*. 2006). All DNA samples were tested for tick mitochondrial 16S recombinant DNA according to published methodology (Black & Piesman 1994; Vial *et al*. 2007) to exclude the occurrence of inhibitors present in the tick homogenates.

### Questionnaire survey

Farm and warthog burrow data were collected from pig farm owners in the surveyed area by an interview-based questionnaire at the same time as the tick sampling was conducted and captured in Microsoft Excel 2010^®^. The questionnaire comprised 23 questions with subcomponents and the collected farm information included main farming activities, use of acaricides, presence of warthogs and other suid species on the farm, contact between warthogs and domestic pigs and estimated number of warthogs and warthog burrows on the farm. The warthog burrow information included individual burrow GPS coordinates, habitat (classified into open veld, bushveld, riverine or wetlands, cultivated lands and others), the soil type (graded as sandy, rocky, muddy and clay), where the burrow was found, whether the burrow was active or inactive and the estimated number of tampans found (many [> 20], few [5–20] and very few [> 5]).

### Spatial distribution

The geographical distribution of the farms, warthog burrows and warthog burrows where tampans were found were mapped using the Geographical Information System software – ArcGIS 10.1 for desktop (ESRI 2012) and DIVA-GIS 7.5.0.0 (Hijmans *et al.* 2012).

### Climate data

The weather data, in monthly averages of minimum and maximum temperatures and millimetres of rainfall and humidity for the period 1993–2012 were obtained from the South African Weather Services in Microsoft Excel 2010^®^. The weather data were summarised into three seasonal averages: summer (December to February), autumn (March to May) and spring (September to November). Winter (June to August) was omitted from the rainfall analysis as the study area is a summer rainfall area with very low potential rainfall in winter. The moving average of these three time periods was calculated using the formula:

$$\text{MA}t=1/n\sum_{j=1}^{i+n-1}at$$ [Eqn 2]

where MA*t* is the moving average at time (*t*), *n* is the number of prior periods to include in the moving average and ‘*at*’ is the actual value at time (*t*). The centred moving average of the two time periods was calculated using the same formula. Linear trend lines (*y* = *mx* + *b*, where *m* is the slope and *b* the intercept) were calculated and plotted using Microsoft Excel 2010 data analysis tools and charts. The linear regression analysis and time series graphs from Microsoft Excel 2010^®^ data analysis tool were used to prove the statistical significance of values.

## Results

### Questionnaire survey results and presence of warthogs and burrows on farms

A much higher proportion of farms had warthog burrows than anticipated when the study was designed. This resulted in the need to sample fewer farms than anticipated in order to meet the required sample size of 61 warthog burrows. Table 1 shows that the required number of warthog burrows sampled exceeded the minimum number required in each province, thus ensuring that the power of the study was met. A total of 73 farms were surveyed in the study area, of which 86.3% had warthog burrows (Table 1) and 72.6% had some sign of warthog activity, with warthogs seen on 66% of the farms during the visit (Table 2). Fifty-eight percent of the farms visited reported seeing warthogs on neighbouring farms. Half the farms visited were wildlife farms, 30.1% farmed with livestock, 11% were crop farmers and the rest were in residential and mixed farming areas. Only one property (a nature reserve) had obvious contact between domestic pigs and warthogs. Eighteen farmers claimed to have seen an increase in the number of warthogs, and this was ascribed to conservation practices on their farms. Although warthogs were not counted, 23.3% of farmers estimated they had between 1 and 20 warthogs on their farms, 17.8% had 21–40, 17.8% had 41–60 and 30.1% had more than 60 (Table 2).

**TABLE 1:** Number of farms and burrows sampled per province and prevalence of Tampans.

Province	Number of farms sampled	Number of farms with burrows	Minimum number of burrows required	Number of burrows sampled	Number of burrows where tampans were found	Number of farms where tampans were found
Gauteng	10	10	5	28	6	4
Limpopo	24	20	15	57	10	7
Mpumalanga	27	24	26	45	3	2
North West	12	9	14	27	1	1

**Total**	**73**	**63**	**61**	**157**†	**20**	**14**

† Includes three storm drains and two nests.

**TABLE 2:** Summary of farm information gathered through questionnaire survey.

Questionnaire variable	Number of samples	Proportion of farms (%)
Provinces sampled	4	-
Farms sampled	73	-
Farms with warthog burrows	63	86
Farming activity, wildlife	37	51
Farming activity, livestock (other than pigs)	22	30
Farming activity, crops	8	11
Mixed farming	3	4
Residential areas	3	4
Farms with tick control practised	28	38
Farms without tick control	45	57
Farms where warthogs were seen during visit	48	66
Farms with warthogs on neighbouring properties	38	52
Farms with estimated number of warthogs 1−20	17	23
Farms with estimated number of warthogs 21−40	13	18
Farms with estimated number of warthogs 41−60	13	18
Farms with estimated number of warthogs > 60	22	30
Farms with an increase in number of warthogs	18	25
Farms with contact between domestic pigs and warthogs	1	1.4

### Presence of tampans and their African swine fever virus infection status along the control line

A total of 152 warthog burrows, three storm drains and two nests were sampled across 63 farms with burrows in the study area (Table 1 and Figure 2). The three storm drains and two nests (flattened grassy areas) were identified by farmers as areas where warthogs were residing on their property, which is why they were included in the sample. Approximately 73.3% of warthog burrows were located in the bushveld area. The sampling teams found 61.8% of burrows were located in sandy soil, 17.2% in muddy soil, 12.7% in clay soil and 8.3% in rocky areas. Out of the sampled warthog burrows, 92% had evidence of active use by animals.

Tampans were recovered from 20 (12.8%) of the sampled warthog burrows (95% confidence interval: 8.0% – 19.0%). Table 1 shows the breakdown of recovered tampans by province. Only 1 out of the 20 burrows from which tampans were recovered had no apparent signs of recent habitation. Approximately 20% of burrows had < 5 tampans, 35% had between 5 and 20 tampans and 45% had more than 20 tampans.

Pools of tampans per burrow were tested for the presence of ASFV DNA. One of the farms, situated along the control line located in Limpopo Province in close proximity to the ASF control line, had tampans in which ASFV DNA was found. However, no live virus was isolated from these PCR-positive tampan samples.

### Climate changes along the control line

**FIGURE 3:**
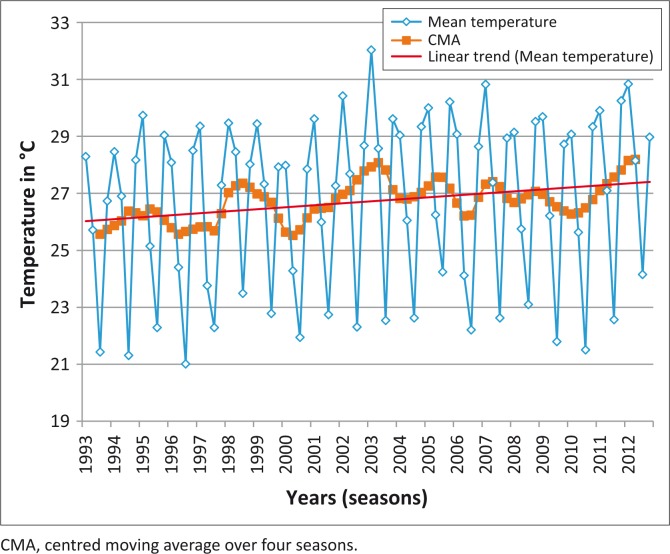
Average seasonal maximum temperature along the African swine fever control line: 1993–2012.

**FIGURE 4:**
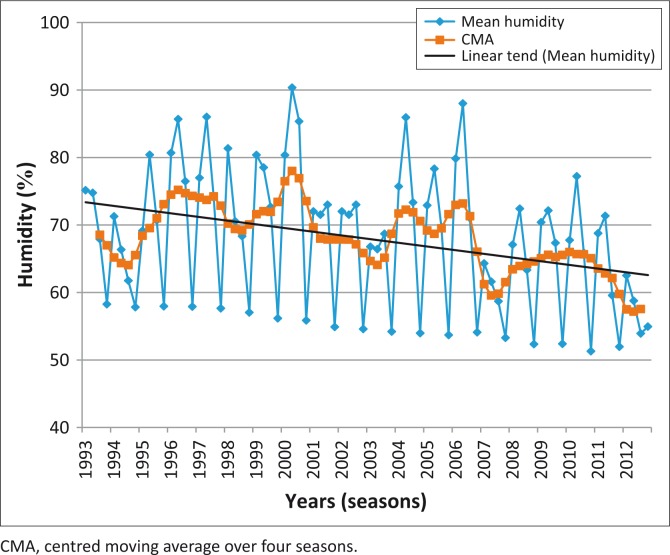
Mean seasonal humidity along the African swine fever control line: 1993–2012.

The data for maximum and minimum average seasonal temperatures showed an increasing trend (Figure 3). On regression analysis, there was a linear increase in maximum temperature between 1995 and 2012 (Linear line; Figure 3) that was statistically significant (*p* = 0.00018), but no significant change in minimum temperature (*p* = 0.6; *r*^2^ = 0.0017). The average humidity in the area along the ASF control line showed a statistically significant decreasing trend (Figure 4) (*p* = 0.003; *r*^2^ = 0.106) at a 95% confidence level, but the decrease observed in seasonal rainfall was not significant (*p* = 0.34; *r*^2^ = 0.015).

## Discussion

Our study confirmed the presence of warthogs along the control line, but it is likely this has been the case throughout the existence of the control line. However, it was not anticipated that such a high proportion of farms would have warthog burrows on them and this impacted on the number of farms finally sampled. The original study design was based on pilot studies carried out in Gauteng Province where at the time approximately 20% of farms had warthog burrows (Pretorius *et al*. 2004). Despite the high prevalence of warthog burrows only 13% of them contained tampans, making the risk of transmission of ASFV along the control line low.

Only 25% of the farmers in our study claimed to have observed an increase in warthog numbers over the past 5 years and this was thought by them to be mainly because of nature conservation practices on their farms. What has changed in recent times along the control line is an increase in game farming in South Africa, including in the areas along the ASF control line. A change from approximately 575 000 to 18.6 million wildlife animals has been documented between 1964 and 2007 (Carruthers 2008), with a threefold increase in the number of wildlife farms between 1981 and 1992. The shift to wildlife-based production has been recognised as the most rapidly expanding agricultural activity (Snijders 2012). The increase in the number of wildlife farms poses a potential risk for movement and increase in the number of warthogs. Studies in the Eastern Cape have shown that common warthogs have the characteristics of an invasive species and have spread beyond the targeted introduction site (Nyafu 2009).

Countering the potential effect of wildlife farms is increasing urban development and market crops along the control line, with farmers in the residential or communal farming areas, mixed farming and crop farming areas claiming that they had observed a decreasing number of warthogs over the years. The main reasons for this decrease were ascribed to hunting, changes in farming practices and changes in human population distribution, with signs of poaching traps identified on some farms. Changes in farming practice towards crop farming could, however, influence the distribution of warthogs because they are known to forage crop fields (FAO 2010) and are likely to gravitate towards cultivated areas, thus increasing the risk of virus being spread along the control line. Expanding communal residential areas, where free-ranging domestic pigs are often kept, could increase the possibility of warthogs and tampans coming into contact with domestic pigs in these areas that could lead to outbreaks.

Adding to the complexity of factors playing a role along the control line is the effect of climate change (Gummow 2010). The study showed that mean daily maximum temperatures have increased and humidity levels have decreased along the control line over the period of the study (1993–2012). If these trends continue, the area along the ASF control line is likely to become dryer and this could also lead to warthogs moving southwards in search of better conditions for survival and thus lead to a wider distribution of tampans as well. Tampans appear to be able to survive drier conditions than those that currently exist along the control line and the change in temperature and humidity is not likely to impact on the survivability of the tampans themselves (D’Huart & Grubb 2001).

The overall infestation rate of the warthog burrows varied amongst provinces and was consistent with what other researchers have found (Bastos *et al.* 2009; Plowright, Parker & Pierce 1969). The study clearly showed that tampan-infested warthog burrows are widely spread throughout the study area both north and south of the ASF control line. The greatest proportion of tampan-infested warthog burrows was in Gauteng Province (21.5% of burrows) and may partly be attributed to targeted sampling based on previous knowledge of where burrows were situated (Pretorius *et al.* 2004). Although ASF DNA was not detected in these tampans, the high proportion of infested burrows in this region supports the need for continued active surveillance along this part of the control line.

In Mpumalanga Province, tampans were found in only 6.67% of warthog burrows on both sides of the control line and no viral DNA was detected by PCR, suggesting that there is probably no need to shift the control line in these areas. Tampans have previously been recorded in the Mpumalanga Province in the area along the Kruger National Park (Penrith *et al.* 2004). Our study mostly found tampans in areas bordering communal residential areas.

The northern portion of the Northwest Province forms part of the ASF-controlled area. The province borders south-western Limpopo Province, which is considered a high-risk area where tampans of *O. moubata* complex are found (Penrith *et al.* 2004). In our study, an insignificant number of tampans (less than 5) was found in one warthog burrow situated north of the ASF control line in Northwest Province and these tested negative for ASFV DNA. Based on the small proportion of infested burrows found in this province, it seems therefore, that there is little need to shift the control line in Northwest Province.

Limpopo Province forms the largest section of the ASF control area where outbreaks of ASF and tampans occur (Penrith *et al.* 2004) and this province had the second highest proportion of tampan-infested burrows (17.5%). It is also the only province to have tampans, which tested positive for ASFV during PCR screening. These were recovered from within a crop farming area situated south of but in close proximity to the ASF control line, suggesting that the control line may need realigning in this area of the country.

From the literature, it would seem that infection rates of tampans with ASFV in the vicinity of the control line have always been low and our study confirms this. Penrith *et al.* (2004) reported rates between 0.3% and 1.7%, whilst Kleiboeker & Scoles (2001) cited rates between 0% and 3.8%. One of the highest infection rates of ASFV reported was in Livingstone Game Park, Zambia, where it was recorded to be 5.1% (Wilkinson *et al.* 1988).

A potential confounder in the study was the stage of ticks collected. Wilkinson *et al.* (1988) emphasised that the overall infection rate of tampans depends on the relative proportions of different stages of ticks with a higher rate where the populations have a higher proportion of adults. The tampans collected for our study had approximately equal proportion of adults and nymphal stages, which may have decreased the sensitivity of detecting virus. Future monitoring may benefit from looking only at adult ticks to detect virus.

## Conclusion

The study confirmed that warthogs, warthog burrows and tampans are found beyond the ASF control line and that regular monitoring of the control line for ASFV is recommended. Only one farm had tampans infected with ASFV, but no live virus was isolated. There is therefore limited evidence of ASFV in tampans outside the ASF control line at this time and the ASF control line remains largely well positioned, with a possible exception in the Limpopo Province.

Changing farming practices to wildlife and crops and changing weather conditions along the control line may be creating environments that are suitable for wider spread of warthogs. This, coupled with increased informal settlement along the control line, could increase the risk of contact with domestic pigs where the virus could be amplified.

Regular monitoring of the control line is therefore recommended and the study serves as a basis for future monitoring.
